# The Burden of Invasive Bacterial Infections in Pemba, Zanzibar

**DOI:** 10.1371/journal.pone.0030350

**Published:** 2012-02-17

**Authors:** Kamala Thriemer, Benedikt Ley, Shaali Ame, Lorenz von Seidlein, Gi Deok Pak, Na Yoon Chang, Ramadhan Hashim, Wolfgang Hellmut Schmied, Clara Jana-Lui Busch, Shanette Nixon, Anne Morrissey, Mahesh K. Puri, Mohammad Ali, R. Leon Ochiai, Thomas Wierzba, Mohammad S. Jiddawi, John D. Clemens, Said M. Ali, Jaqueline L. Deen

**Affiliations:** 1 International Vaccine Institute, Seoul, Republic of Korea; 2 University of Vienna, Biocenter, Vienna, Austria; 3 Public Health Laboratory (Pemba) – Ivo de Carneri, Chake Chake, Tanzania; 4 Ministry of Health and Social Welfare, Zanzibar, Tanzania; 5 Menzies School of Health Research, Casuarina, Northern Territory, Australia; 6 Institute of Molecular Biotechnology GmbH, Vienna, Austria; 7 Duke University-KCMC Collaboration, Moshi, Tanzania; Rockefeller University, United States of America

## Abstract

**Background:**

We conducted a surveillance study to determine the leading causes of bloodstream infection in febrile patients seeking treatment at three district hospitals in Pemba Island, Zanzibar, Tanzania, an area with low malaria transmission.

**Methods:**

All patients above two months of age presenting to hospital with fever were screened, and blood was collected for microbiologic culture and malaria testing. Bacterial sepsis and malaria crude incidence rates were calculated for a one-year period and were adjusted for study participation and diagnostic sensitivity of blood culture.

**Results:**

Blood culture was performed on 2,209 patients. Among them, 166 (8%) samples yielded bacterial growth; 87 (4%) were considered as likely contaminants; and 79 (4%) as pathogenic bacteria. The most frequent pathogenic bacteria isolated were *Salmonella* Typhi (n = 46; 58%), followed by *Streptococcus pneumoniae* (n = 12; 15%). The crude bacteremia rate was 6/100,000 but when adjusted for potentially missed cases the rate may be as high as 163/100,000. Crude and adjusted rates for *S.* Typhi infections and malaria were 4 and 110/100,000 and 4 and 47/100,000, respectively. Twenty three (51%), 22 (49%) and 22 (49%) of the *S.*Typhi isolates were found to be resistant toward ampicillin, chloramphenicol and cotrimoxazole, respectively. Multidrug resistance (MDR) against the three antimicrobials was detected in 42% of the isolates.

**Conclusions:**

In the presence of very low malaria incidence we found high rates of *S.* Typhi and *S. pneumoniae* infections on Pemba Island, Zanzibar. Preventive measures such as vaccination could reduce the febrile disease burden.

## Introduction

For decades, falciparum malaria has been the leading cause of febrile illness among patients in sub-Saharan Africa presenting to hospitals for treatment. However, during the last ten years, there has been a decline in malaria in many parts of sub-Saharan Africa [1], [2], [3], [4]. Little information exists about the current leading causes of severe febrile illness in areas of sub-Saharan Africa where malaria control strategies have been deployed. Limited diagnostic facilities have led to a lack of data on community-acquired invasive bacterial infection [5]. Recent reviews have tried to overcome this problem by aggregating available information on bloodstream infections [6] and extrapolating incidence rates [7]. But these are limited by the diversity of the continent and its populations. More site-specific and detailed information on invasive bacterial infections is required to improve the prevention and management of febrile illnesses in the region.

Following the deployment of artemisinin-based combination therapy starting in late 2003 and the programmatic distribution of long-lasting insecticidal nets from early 2006 in Zanzibar, Tanzania, malaria-associated morbidity and mortality has decreased dramatically [8], [9]. We conducted a surveillance study to determine the leading causes of bloodstream infections in febrile patients seeking treatment at district hospitals in Pemba Island, Zanzibar, and calculated incidence rates for the major pathogens isolated.

## Materials and Methods

### Ethics

The study was conducted according to the principles expressed in the Declaration of Helsinki. Written informed consent was obtained from all study participants or their legal guardians. The Zanzibar Medical Research and Ethics Committee and the International Vaccine Institute - Institutional Review Board in South Korea approved this project.

### Study site and population

**Figure 1:**
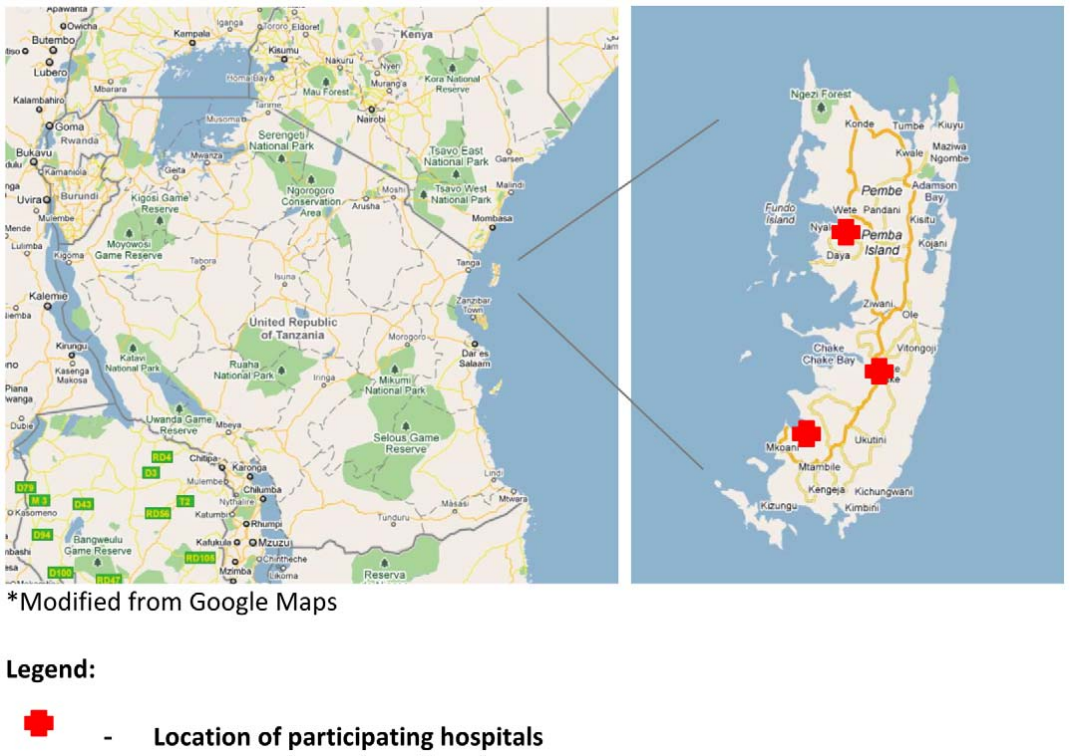
Study site.

The study was conducted on Pemba, one of the main islands of the Zanzibar archipelago (Figure 1). The total area of Pemba is approximately 984 square kilometres and much of its terrain is hilly, heavily vegetated, and poorly accessible through mainly unpaved roads. The northern region of Pemba is divided into Micheweni and Wete districts, while the southern region is divided into Mkoani and Chake-Chake districts. The administrative center is Chake-Chake, which is located in the district of the same name. In 2010, the population of Pemba was estimated to be 500,600 with more than 50% of the population below the age of 15 years [10], [11].

Pemba is mainly rural, with an economy based on fishing, seaweed harvesting, and the farming of cloves, coconut, and copra. Primary health care is delivered through 58 primary health care units (PHCU) that provide minor services and can admit one to two inpatients. The units have limited dispensaries but usually have no doctors or laboratory facilities. Patients that require secondary health care are referred to one of the three district hospitals: Chake Chake, Wete, or Mkoani. Additionally, there are the Vitongoji and Micheweni cottage hospitals, which are mainly used during outbreaks of diarrheal diseases.

The Zanzibari Expanded Programme of Immunization (EPI) includes the following vaccines: Bacille Calmette-Guérin (BCG), live oral polio, diphtheria-whole cell pertussis-tetanus-hepatitis B, and the monovalent measles vaccines for children, as well as the supplemental tetanus toxoid vaccine for women of child-bearing age. *Haemophilus influenzae* type b and pneumococcal vaccines are currently not included in the EPI. Typhoid vaccine is not routinely administered in Zanzibar.

### Study procedures

The study was implemented in phases. Surveillance was started first at Chake Chake District Hospital in March 2009, followed by Mkoani District Hospital in May 2009, and Wete District Hospital in August 2009 (Figure 1). Surveillance was continued in all three hospitals until the end of December 2010. Clinical information was recorded on a standard digital case record form using handheld computers, also known as personal digital assistants (PDAs) [12]. Treatment was provided for patients according to national guidelines.

Patients presenting for care during the study period were screened for eligibility. Subjects above the age of 2 months with a recorded temperature of ≥37.5°C (tympanic thermometer) presenting at the outpatient department (OPD) or subjects above the age of 2 months admitted to a hospital with any history of fever were included in the study. Study staff screened all patients at the OPD during opening hours from 08:00 to 13:00, Monday to Friday. Eligible patients were referred to the study nurse for informed consent, enrolment and blood collection, while waiting to see the hospital health worker who prescribed additional diagnostic tests and treatment, or admitted the patient to the ward. Patients who did not present with a documented temperature of ≥37.5°C but with a history of fever were later enrolled into the study if admitted to the ward. Enrolment of inpatients into the study was conducted in the afternoon after closure of the OPD. Patients admitted during the early evening and night, were screened for eligibility the following morning.

### Point-of-care and laboratory investigations

We collected 1 to 8 millilitres of blood from children and 9 to 12 millilitres from adults. Immediate bedside testing was done for hemoglobin and glucose (Hemocue, Anlkholm Sweden). About 1–3 ml of blood from children and 8–10 ml from adults were used to inoculate pediatric and adult BACTEC bottles (Peds Plus Aerobic and Plus Aerobic; BD, USA), respectively, and incubated in an automated blood culture machine (BACTEC 9050, BD, USA). Blood cultures were processed according to standard guidelines [13]. Api20E (Biomerieux, France) was used for further identification of gram-negative rods, which was followed by serological confirmation for all presumptive *Salmonella* isolates. The remaining blood samples were used for malaria rapid diagnostic testing (Paracheck, Orchid Biomedical, Mumbai, India) and the preparation of thin and thick blood films. The films were Giemsa-stained and read. At least 300 high power microscopic fields were examined to exclude malaria.

Antimicrobial susceptibility testing was performed using the modified Kirby-Bauer disc diffusion method and the mean inhibitory concentration was assessed using Etest. Evaluation was done according to the guidelines of the Clinical and Laboratory Standards Institute [14].

### Data management, definitions and analysis

Clinical data entered directly into PDAs had real-time error, range and consistency checks, and were transferred to the central database at regular intervals [12]. HIV test results were obtained from hospital records if the patient consented, and were entered into the database with no patient identifier information. Laboratory results were recorded on paper forms and double-entered into custom-made data entry programs using FoxPro software (Microsoft, Seattle, Washington, USA). The de-identified clinical data were linked to the laboratory results, using each participant's study identification number.

The following definitions were applied during the analysis. Bacteremia was defined as fever with isolation of pathogenic bacteria from blood culture. Contaminants were defined as non-pathogenic bacteria most probably derived from the skin during blood collection and included *Bacillus* spp., *Corynebacterium* and coagulase-negative *Staphylococci*. Malaria was defined as fever with a positive blood film and/or positive rapid diagnostic test (RDT).

In order to avoid seasonal confounding in the incidence rates, we used data from January to December 2010 only. Incidence rates were calculated per 100,000 persons and by age group: ≤5, >5–15 and >15 years old. The total and age-specific number of cases was used as the numerator and the respective population for 2010 projected from the 2002 census [10], [11] as the denominator. In the primary crude analysis we assumed that the surveillance system captured all patients in Pemba with severe febrile illness who presented to hospital from January to December 2010. Crude incidence rates and their 95% confidence intervals (CI) were calculated using the Wilson score method. Data on health- seeking behaviour from four areas around Chake Chake District Hospital showed an average use of the hospital of 10.6% for febrile patients (Kaljee and Pach, unpublished data). In a sensitivity analysis for bacteremia, crude rates were multiplied by a factor of 9.43 (1/health care utilisation rate) to adjust for missed cases due to health seeking behaviour, by a factor 1.36 (1/[1- proportion non enrolled]) to adjust for those eligible but not proceeding to enrolment (26.4%), by factor 1.02 and 1.04 (1/[1- proportion not consented and insufficient blood]) to adjust for those not consenting or were insufficient amounts of blood were collected (2.42%, 4.01%, 1.85% and 1.50% depending on age group), as well as by a factor of 2 (1/[1- culture sensitivity]) for an average blood culture sensitivity of 50% [7], [15], [16], [17], [18]. In the sensitivity analysis for malaria, crude incidence rates were multiplied by the same factors as the bacteremia results to adjust for health seeking behaviour (factor 9.43) and for the proportion of eligible patients that were not enrolled (factor 1.36). In addition malaria crude incidence rates were multiplied by a factor of 1.02, 10.4 and 10.1 depending on age group to adjust for the proportion of patients that did not consent or had no malaria test done (total: 2.42%; ≤5 years: 4.29%; >5–15 years: 1.51% and >15 years: 1.44%).

All analyses were performed using Microsoft Excel spreadsheets and Stata version 10 (StataCorp, College Station, TX, USA).

## Results

**Figure 2:**
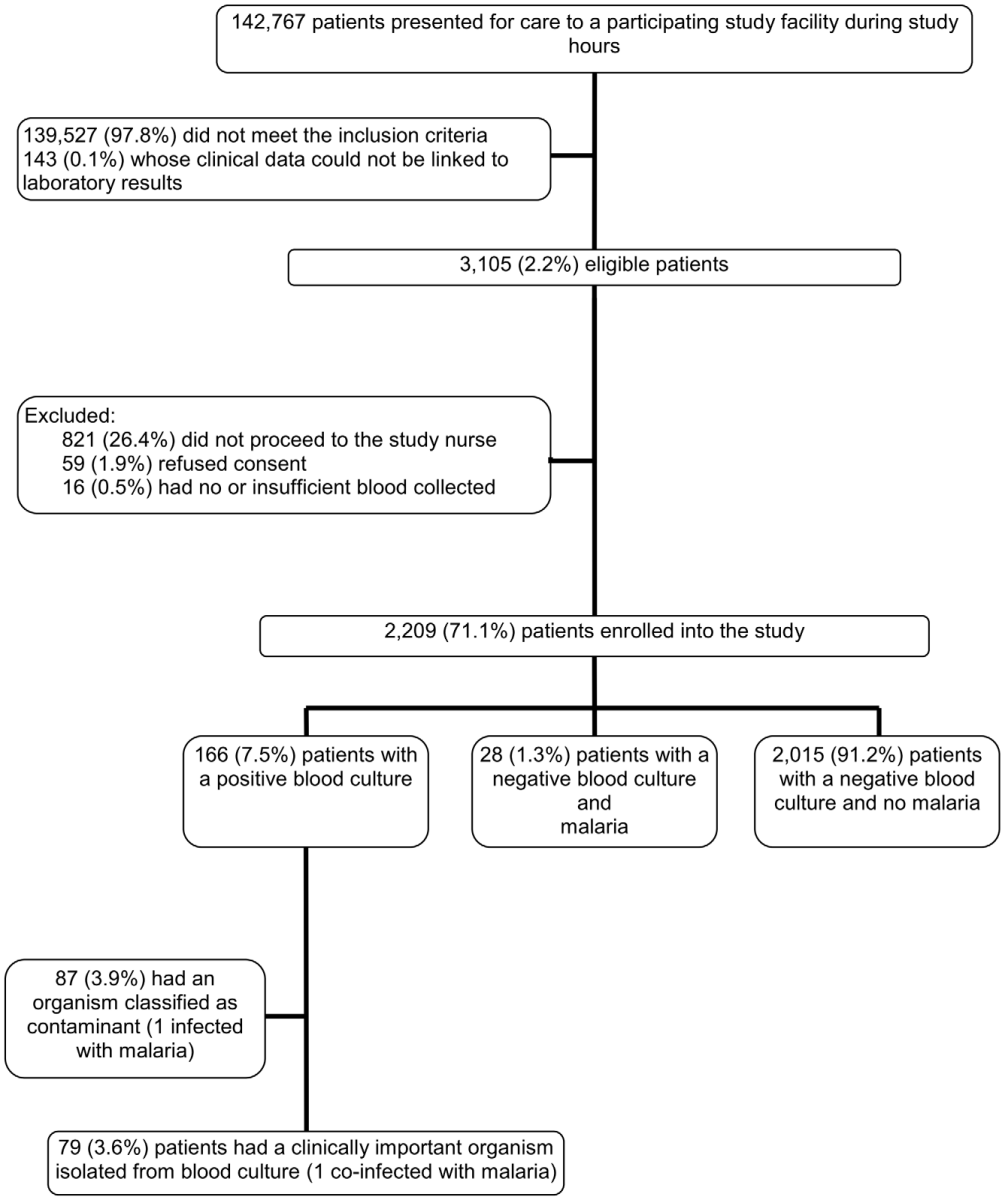
Assembly of patients in the study.

A total of 142,767 patients presented for care during study hours at the three participating hospitals and were screened for eligibility (Figure 2). Of these, 3,105 (2%) patients were found to be eligible. There were 821 (26%) who did not proceed to the study nurse, 59 (2%) who refused participation, and 16 (0.5%) from whom insufficient amounts of blood were drawn. There were no significant differences (p>0.05) regarding sex, age or temperature among eligible patients enrolled and eligible patients not enrolled. Blood culture was performed on 2,209 (71%) patients and these results were included in the analysis. Of these, 166 (8%) samples yielded bacterial growth: 87 (4%) were considered as likely contaminants and 79 (4%) as pathogenic bacteria. Among the 2,209 patients, RDT for malaria was done for 2,193 (99%) cases and blood films for 2,147 (97%) cases. One (0.04%) patient had a co-infection of bacteremia and malaria (*Plasmodium falciparum*), whereas 28 (1%) had malaria (*P. falciparum*) with a negative blood culture (Figure 2). Only one patient agreed to be tested for HIV and two patients reported to have had tests done previously.

We assessed the bacteremia cases by hospital (Table 1). Of the 2,209 patients, 975 (44%) were enrolled at Chake Chake District Hospital from 16th March 2009 to 30th December 2010; 682 (31%) at Mkoani District Hospital from 4th May 2009 to 30th December 2010; and 552 (25%) at Wete District Hospital from 5th August 2009 to 30th December 2010. The case fraction of bacteremia varied significantly (p<0.01) from 2% to 5% across the sites. Overall, the most frequent pathogenic species isolated was *Salmonella* Typhi (n = 46; 58%).

**Table 1:** Number (%) of bacteremia cases by participating hospital during the entire study period (2009–2010).

	Chake Chake	Mkoani	Wete	Total	p
	n (%)	n (%)	n (%)	n (%)	
Patients enrolled	975	682	552	2209	
Period of surveillance	16/03/2009–30/12/2010	04/05/2009–30/12/2010	05/08/2009–30/12/2010		
Pathogenic bacteria isolated	30 (3.1%)	37 (5.4%)	12 (2.2%)	79 (3.6%)	0.005
*S.*Typhi	20	21	5	46	0.3
	(66.7%)	(56.8%)	(41.7%)	(58.2%)	
*S.pneumoniae*	2	5	5	12	0.02
	(6.7%)	(13.1%)	(41.7%)	(15.2%)	
*E.coli*	4	1	0	5	0.17
	(13.3%)	(2.7%)	(0%)	(6.3%)	
*S.aureus*	3	1	1	5	0.36
	(10%)	(2.7%)	(8.3%)	(6.3%)	
Hib	1	2	0	3	1.0
	(3.3%)	(5.4%)	(0%)	(3.8%)	
Others	0	7	1	8	0.03
	(0%)	(28.9%)	(8.3%)	(10.1%)	

Of the 2,209 patients included in the analysis, 637 (29%) were ≤5 years old, 490 (22%) were >5 to 15 years old, and 1,082 (49%) were >15 years old (Table 2). The pathogenic bacterial isolates were ranked according to frequency and by age group (Table 2). *S.* Typhi was found to be the leading pathogen in all age groups, causing 10/29 (35%), 12/16 (75%) and 24/34 (71%) of bacteremia, respectively and 46 cases (58%) overall. The second most frequently isolated pathogen was *Streptococcus pneumoniae*, which was responsible for 12 (15%) bacteremia cases overall; and 8/29 (28%) among those ≤5 years old, 3/16 (19%) among those 5 to 15 years of age and 1/34 (3%) among those >15 years. Other commonly isolated bacterial pathogens were *Escherichia coli*, *Staphylococcus aureus*, and *Haemophilus influenza*e type b (Hib).

**Table 2:** Age-specific ranking of pathogens during the entire study period.

	≤5 y(n = 637)	Rank	>5 y–15 y (n = 490)	Rank	>15 y (n = 1082	Rank	Total no (%)	Overall rank
All pathogenic bacteria	29		16		34		79	
- *Salmonella* Typhi	10	1	12	1	24	1	46	1
	(34.5%)		(75%)		(70.6%)		(58.2%)	
- *S.pneumoniae*	8	2	3	2	1	4	12	2
	(27.6%)		(18.7%)		(2.9%)		(15.2%)	
- *E.coli*	0		0		5	2	5	3
	(0%)		(0%)		(14.7%)		(6.3%)	
- *S.aureus*	2	3	1	3	2	3	5	3
	(6.9%)		(6.2%)		(5.9%)		(6.3%)	
- Hib	2	3	0		1	4	3	4
	(6.9%)		(0%)		(2.9%)		(3.8%)	
- other	7		0		1		8	–
	(24.1%)		(0%)		(2.9%)		(10.1%)	
Contaminants	52		5		30		87	
Total	81		21		64		166	

We calculated the crude and adjusted incidence rates for bacteremia (Table 3) and falciparum malaria (Table 4). The crude incidence rate for invasive bacteremia was 6 per 100,000 (95%CI: 4 to 9) persons per year (Table 3). Adjustments yielded an incidence rate of 163/100,000 (95%CI: 152 to 174) with a rate of 146/100,000 (95%CI: 127 to 169) for children less than 6 years old, 162/100,000 (95%CI: 142 to 186) for those 6 to 15 years old and 171/100,000 (95%CI; 155 to 188) for those above 15 years of age. No significant differences between age groups were found (p>0.05).

**Table 3:** Crude and adjusted incidence rates for bacteremia.

	No. of cases (2010)	Population in catchment area 2010*	Crude incidence rate per 100,000 (95%CI)	p	Health seeking behaviour (%) **	Adjusted rate I	Did not proceed to study nurse (%)	Adjusted rate II	No consent; insuff. blood (%)	Adjusted rate III	Blood culture sensitivity (%)***	Adjusted incidencerate per 100,000(95% CI)
**Pathog. bacteria total**	31	500600	6 (4 to 9)		10.6	58	26.4	79	2.42	81	50	163(152 to 174)
**Pathog. bacteria ≤5 years**	7	127848	5 (3 to 11)		10.6	52	26.4	70	4.01	73	50	146(127 to 169)
**Pathog. bacteria >5–15 years**	8	128917	6 (3 to 12)	0.923	10.6	59	26.4	80	1.85	81	50	162(142 to 186)
**Pathog. bacteria >15 years**	16	243835	7 (4 to 11)		10.6	62	26.4	84	1.50	85	50	171(155 to 188)
***S.*** ** Typhi** **total**	21	500600	4 (3 to 6)		10.6	40	26.4	54	2.42	55	50	110(102 to 120)
***S.*** ** Typhi** **≤5 years**	4	127848	3 (1 to 8)		10.6	30	26.4	40	4.01	42	50	84(69 to 101)
***S.*** ** Typhi >5–15 years**	5	128917	4 (2 to 9)	0.710	10.6	37	26.4	50	1.85	51	50	101(86 to 121)
***S.*** ** Typhi >15 years**	12	243835	5 (3 to 9)		10.6	46	26.4	63	1.50	64	50	128(115 to 143)
***S. pneumoniae*** ** total**	4	500600	1 (0.3 to 2)		10.6	8	26.4	10	2.42	11	50	21(17 to 25)
***S. pneumoniae*** ** ≤5 years**	2	127848	2 (0.4 to 6)		10.6	15	26.4	20	4.01	21	50	42(32 to 54)
***S. pneumoniae*** ** >5–15 years**	2	128917	2 (0.4 to 6)	0.150	10.6	15	26.4	20	1.85	20	50	41(31 to 53)
***S. pneumoniae*** ** >15 years**	0	243835	0 (0 to 2)		10.6	0	26.4	0	1.50	0	50	0(0 to 2)
***E. coli*** ** total**	3	500600	1 (0.2 to 2)		10.6	6	26.4	8	2.42	8	50	16(13 to 20)
***S.aureus*** ** total**	2	500600	0		10.6	4	26.4	5	2.42	5	50	11(8 to 14)

* According to projections of the national census 2002;

** People attending Chake Chake Hospital by %; Kaljee and Pach, unpublished data;

*** According to Crump *et al*, 2004; Zhou & Pollard 2010, Wain *et al.* 2011 and 2008; Saha *et al.*, 2010.

**Table 4:** Crude and adjusted incidence rates for malaria.

	No. of cases in 2010 only	Population in catchment area 2010*	Crude incidence rate per 100,000 (95%CI)	p	Health-seeking behaviour (%)**	Adjusted rate I	Did not proceed to study nurse (%)	Adjusted rate II	No consent;no malariatest (%)	Adjusted incidence rate per 100,000 (95%CI)
**Malaria (RDT and/or slide pos.) total**	18	500600	4 (2 to 6)		10.6	34	26.4	46	2.42	47 (42 to 54)
**Malaria (only slide) total**	3	500600	1 (0.2 to 2)		10.6	6	26.4	8	2.42	8 (6 to 11)
**Malaria (RDT and/or slide pos.) ≤5 years**	10	127848	8 (4 to 14)		10.6	74	26.4	100	4.29	105 (89 to 124)
**Malaria (RDT and/or slide pos.) 5–15 years**	5	128917	4 (2 to 9)	0.006	10.6	37	26.4	50	1.51	51 (40 to 64)
**Malaria (RDT and/or slide pos.) >15 years**	3	243835	1 (0.4 to 4)		10.6	12	26.4	16	1.44	16 (12 to 22)

* According to projections of the national census 2002;

** People attending Chake Chake Hospital by %; Kaljee and Pach, unpublished data.

The leading pathogen, *S.* Typhi, had a crude incidence rate of 4/100,000 (95%CI: 3 to 6) and an adjusted rate of 110/100,000 (95%CI: 102 to 120). The age-specific incidence rates of typhoid fever increased with age (Table 3). *S. pneumoniae* rates were highest among children below 15 years of age (Table 3). The crude invasive bacteremia rates in female patients and male patients were 7/100,000 (95%CI: 4 to 11) and 6/100,000 (95%CI: 3 to 10), respectively. The crude typhoid fever rate in females was 5/100,000 (95%CI: 3 to 9) and in males was 3/100,000 (95%CI: 2 to 6) (p>0.05).

The overall crude incidence rate of falciparum malaria was 4/100,000 (95%CI: 2 to 6) and 1/100,000 (95%CI: 0.2 to 2) if only slide-positive cases were included in the analysis (Table 4). Adjustments yielded rates of 47/100,000 (95%CI: 42 to 54) and 8/100,000 (95%CI: 6 to 11), respectively. Age-specific adjusted rates were highest in the youngest age group and showed statistical difference (p<0.01) (Table 4).

Antibiotic susceptibility was assessed for 45/46 (98%) *S.*Typhi isolates (Table 5) and for all *S. pneumoniae*, *E. coli*, *S.aureus*, and Hib isolates (Table 6). Twenty three (51%), 22 (49%) and 22 (49%) of the *S.*Typhi isolates were found to be resistant to ampicillin, chloramphenicol and cotrimoxazole, respectively. One isolate was found to be resistant to ciprofloxacin. Multidrug resistance (MDR) against the three first-line antimicrobials was detected in 19/45 (42%) isolates. Out of these 19 MDR isolates, four (21%) were resistant to nalidixic acid, but none of them was resistant to ciprofloxacin.

**Table 5:** Susceptibility patterns of *S.*Typhi (n = 45)*.

	Resistant	Intermediate	Susceptible
	n (%)	n (%)	n (%)
Ampicillin	23 (51.1%)	2 (4.4%)	20 (44.4%)
Chloramphenicol	22 (48.9%)	2 (4.4%)	21 (46.7%)
Trimethoprim-sulfamethoxazole (Cotrimoxazol)	22 (48.9%)	1 (2.2%)	22 (48.8%)
Amoxicillin/Clavulanic acid	0 (0%)	1 (2.2%)	44 (97.8%)
Cefazolin	2 (4.4%)	3 (6.7%)	40 (88.9%)
Ceftazidime	3 (6.7%)	0 (0%)	42 (93.3%)
Ceftriaxon	0 (0%)	0 (0%)	45 (100%)
Ciprofloxacin	1 (2.2%)	0 (0%)	44 (97.8%)
Gentamycin	3 (6.7%)	1 (2.2%)	41 (92.1%)
Nalidixic acid	14 (31.1%)	2 (4.4%)	29 (64.4%)
Ampicillin + Chloramphenicol + Trimethoprim-sulfamethoxazole	19 (42.2%)	0 (0%)	19 (42.2%)
Ampicillin + Chloramphenicol + Trimethoprim-sulfamethoxazole + Nalidixic acid	4 (8.9%)	0 (0%)	9 (20%)
Ampicillin + Chloramphenicol + Trimethoprim-sulfamethoxazole + Nalidixic Acid + Ciprofloxacin	0 (0%)	0 (0%)	9 (20%)

* According to *Performance Standards for Antimicrobial Susceptibility Testing*, M100-S16, Vol 26. No. 3. CLSI, January 2007.

**Table 6:** Susceptibility patterns of *S.pneumoniae, E.coli, S.aureus* and Hib*.

	Resistant	Intermediate	Susceptible
*S. pneumoniae* (n = 12)	n (%)	n (%)	n (%)
Chloramphenicol	0 (0%)	0 (0%)	12 (100%)
Erythromycin	0 (0%)	0 (0%)	12 (100%)
Trimethoprim/sulfadoxin	7 (58.3%)	2 (16.7%)	3 (25%)
Penecillin	3 (25%)	0 (0%)	9 (75%)

*E.coli* (n = 5)			
Amoxicillin/Clavulanic acid	0 (0%)	2 (40%)	3 (60%)
Ampicillin	3 (60%)	0 (0%)	2 (40%)
Cefazolin; Ceftazidime; Ceftrioxone	0 (0%)	0 (0%)	5 (100%)
Chloramphenicol; Ciprofloxacine; Gentamycin	1 (20%)	0 (0%)	4 (80%)
Nalidixic acid	2 (40%)	0 (0%)	3 (60%)
Trimethoprim/sulfadoxin	4 (80%)	0 (0%)	1 (20%)

*S.aureus* (n = 5)			
Cefoxitin (Oxacillin); Vancomycin	0 (0%)	0 (0%)	5 (100%)
Chloramphenicol; Erythromicin	0 (0%)	1 (20%)	4 (80%)
Penicillin	4 (80%)	0 (0%)	1 (20%)
Trimethoprim/sulfadoxin	2 (40%)	0 (0%)	3 (60%)

Hib (n = 3)			
Ampicillin	2 (66.7%)	0 (0%)	1 (33.3%)
Ceftriaxone	0 (0%)	0 (0%)	3 (100%)
Chloramphenicol; Trimethoprim/sulfadoxin	3 (100%)	0 (0%)	0 (0%)

* According to *Performance Standards for Antimicrobial Susceptibility Testing*, M100-S16, Vol 26. No. 3. CLSI, January 2007.

## Discussion

We found that *S.* Typhi to be the leading pathogen in adults and children presenting to hospitals with severe febrile illness in an area of low malaria transmission. Our adjusted rate of 110 typhoid fever cases per 100,000 population falls into the category of “high typhoid incidence area (>100 per 100,000 population per year)” based on the classification by Crump *et al.* [7]. In accordance with earlier findings [6], the incidence rates of blood culture-confirmed typhoid fever cases increased with age. Higher rates were also found among the female population, compared with the male population, though not at a statistically significant level. *S. pneumoniae* was the second most frequently isolated pathogen in all age groups.

Incidence data on community-acquired bacteremia from this region is sparse. A study from 2006 from western Kenya reports incidence rates of bacteremia among children below the age of 2 and below 5 years to be 1,741 and 909 per 100,000 children, respectively [19], considerably higher than our findings. However, the Kenya study was a community-based study that included a demographic surveillance system, which allowed for a more complete detection of cases than our hospital-based sentinel study. Site-specific differences in malaria transmission may also influence the rates and etiology of invasive bacteremia. Since malaria has been shown to predispose to bacteremia [20], it is likely that the main reason for the lower positivity rate is the low malaria rate in Pemba. The dramatic reduction of malaria transmission in Zanzibar [9], may therefore provide additional benefits for the local population. This is supported by studies that showed that the control of falciparum malaria in Zanzibar was associated with a decrease in childhood mortality between 2002 and 2005 [8]. In our study we did not isolate any non-typhoidal *Salmonella*. Falciparum malaria is associated with invasive non-typhoidal *Salmonellosis* and also likely accounts for these differences [21].

Reddy *et al.* [6] conducted a recent review of studies that used blood culture to identify non-malaria bloodstream infections among patients admitted to hospitals in Africa. Based on 22 studies from 1984 to 2006 and on a total of 58,296 patients, the leading bacterial isolate was *S. enterica* (1,643 or 29.1%). Fifty eight percent of these were non-typhoidal *Salmonella*, 34% were *S.* Typhi, and 8% were unspecified S. *enterica* isolates. A recent study by Crump *et al* found *S.*Typhi the leading pathogen among HIV negative children in the Moshi area with 6 out of 341 patients [22].

We found significant differences in the isolation rate of pathogenic bacteria among hospitals. Potential explanations for this phenomenon might be differences in health-seeking behaviour. The differences were not due to outbreaks since the cases were rather evenly distributed over the entire study period.

With the exception of *S.* Typhi, all isolates were found to be susceptible to at least one locally available antibiotic. About half of the *Salmonella* Typhi isolates were resistant to at least one of the frequently used drugs: ampicillin, chloramphenicol, and trimethoprim-sulfadoxine. More than 40% showed resistance toward all three drugs (multi-drug resistance). These are considerably higher resistance rates than those reported between 1995 and 2008 [6]. However, more recent data from Kenya showed even higher numbers of resistant isolates [23] and reports from Kenya warn about rising resistance towards fluoroquinolones [24]. Although we found low resistance towards ciprofloxacin, continued monitoring of susceptibility patterns is essential.

The current routine treatment for inpatients with suspected bacteremia is ampicillin and gentamicin. If this fails and subject to availability ceftriaxone is given. For outpatients ciprofloxacin is routinely given if bacteremia is suspected. According to our data this strategy seems appropriate especially since full susceptibility towards ceftriaxone was found for S.Typhi, E.coli and Hib.

This study had several limitations. Firstly, incidence calculations were adjusted for health-seeking behaviour based on findings from four villages around Chake Chake District Hospital only (Kaljee and Pach, unpublished). Variations in health-seeking behaviour by geographic distance to the hospital, sex, or age were not taken into account. It is possible that a larger percentage than estimated make use of the participating health care facilities during a prolonged course of disease, if not as the first choice, then as a later alternative. Secondly, the study period was brief and no changes in disease trends could not be detected. Thirdly, only single blood cultures were collected. Repeated sample collection and multiple cultures from each patient might have increased the overall positivity rate [25]. Also greater blood volumes may have increased the isolation rate but this would have been difficult toe carry-out. Finally, there was very limited information on the HIV status of patients. We were unable to compare incidence of bloodborne pathogens between HIV-positive and HIV-negative patients. Previous HIV prevalence studies in Zanzibar have shown an infection rate in the general population on both islands Pemba and Unguja to be 0.6% in 2003 [26], suggesting a limited impact on our findings.

In summary, in the presence of very low malaria incidence we found high rates of typhoid fever in all age groups and *S. pneumoniae* among children on Pemba Island, Zanzibar. The data indicate the need to consider preventive strategies against these diseases to further decrease the burden of severe febrile illness. These include long-term improvements in education, hygiene behaviour such as hand-washing, water supply sanitation to address the high *S.*Typhi incidence. In addition vaccination programmes against typhoid fever and the introduction of the pneumococcal vaccine in the EPI program deserves consideration.
